# COVID-19 Effects on Livestock Production: A One Welfare Issue

**DOI:** 10.3389/fvets.2020.585787

**Published:** 2020-09-30

**Authors:** Jeremy N. Marchant-Forde, Laura A. Boyle

**Affiliations:** ^1^United States Department of Agriculture - Agricultural Research Service, Livestock Behavior Research Unit, West Lafayette, IN, United States; ^2^Pig Development Department, Teagasc Animal and Grassland Research and Innovation Centre, Fermoy, Ireland

**Keywords:** poultry, pigs, livestock production chain, one welfare, COVID-19

## Abstract

The COVID-19 pandemic highlights that we exist in a global community. From a single city, it spread to 188 countries across the world and infected 30 million people by September 18, 2020. Decades of modeling pandemics predicted potential consequences, but COVID-19's impact on the food supply chain, and specifically livestock production was unexpected. Clusters of cases among workers in meat processing plants evolved quickly to affect human, animal, and environmental welfare in several countries. In processing plants, the hygiene focus is on product quality and food safety. Because of their close proximity to one another, COVID-19 spread rapidly between workers and the lack of sick leave and health insurance likely resulted in workers continuing to work when infectious. In the United States (U.S.) many processing plants shut down when they identified major outbreaks, putting pressure especially on pig and poultry industries. At one point, there was a 45% reduction in pig processing capacity meaning about 250,000 pigs per day were not slaughtered. This resulted in longer transport distances to plants in operation with extra capacity, but also to crowding of animals on farm. Producers were encouraged to slow growth rates, but some had to cull animals on farm in ways that likely included suffering and caused considerable upset to owners and workers. Carcass disposal was also associated with potential biosecurity risks and detrimental effects on the environment. Hence, this is a One Welfare issue, affecting human, animal, and environmental welfare and highlighting the fragility of intensive, high-throughput livestock production systems. This model needs to be re-shaped to include the animal, human, and environmental elements across the farm to fork chain. Such a One Welfare approach will ensure that food production systems are resilient, flexible, and fair in the face of future challenges.

## Introduction

The emergence of a novel pandemic disease should not have taken the world by surprise. Within the last century, the 1918 influenza pandemic infected an estimated 500 million people and killed 17–50 million (1). More recently, the 2009 swine flu pandemic infected about 61 million and killed an estimated 284,000 (2). Both pandemics were H1N1 influenza viral diseases and it is perhaps natural that the focus for predicting future pandemics was on influenza, with a Web of Science search for “pandemic AND prediction” showing that 290 out of 415 articles since 2010 include “influenza” [e.g., (3)] whereas only 15 include “coronavirus” [e.g., (4)]. However, recent SARS and MERS outbreaks showed that coronaviruses are strong candidates for zoonotic pathogen spillover (5). This is combined with the threat of zoonoses emerging from wild animal populations, especially in regions of the world where wildlife biodiversity is high and land-use change is occurring (6). This is against a background of pressures arising from climate change, food security, and safety (7) and antimicrobial use and resistance (8).

After the swine flu pandemic, the World Health Organization conducted a review of its first line of defense—its International Health Regulations (2005)—and concluded that, “The world is ill-prepared to respond to a…global, sustained and threatening public-health emergency.” (9). Until now, the major perceived threats in intensive livestock production were a pandemic outbreak of a foreign animal viral disease, exacerbated by secondary bacterial infections and potential concurrent antimicrobial resistance driven by use of medically important antimicrobials. A pandemic may bring expected challenges but there are always unforeseen ramifications that transcend human health (10). The interconnectivity of human health with that of animals and the environment is captured in the One Health concept, which is defined as “the collaborative efforts of multiple disciplines working locally, nationally, and globally, to attain optimal health for people, animals, and our environment” (11). The concept of One Welfare extends One Health to recognize “the interconnections between animal welfare, human well-being and the environment” (12). This paper will focus on the impact that COVID-19 is having on One Welfare within livestock production from farm to fork with particular focus on the pig and poultry industries. We focus on the United States (U.S.) as it is one of the hardest hit countries so far where related data are readily available and accessible. However, we expect that the situation is similar in all affected countries with intensive livestock production industries.

## COVID-19 Effects on the Livestock Product Supply Chain

Livestock, and particularly pig and poultry, production in the industrialized world, and increasingly in the developing world, is characterized by its intensive nature, initially driven by post-war government policies intended to increase production and decrease cost, but now sustained by consumer demand for cheap food (13). Farms are fewer in number but larger, with more animals and birds per holding in enclosed, climate-controlled buildings, with more automation and fewer stockpersons. Vertical integration is common, meaning a single company will own all parts of the system, from feed mill to processing plant. The production system is primed for maximum output, with all parts of the chain from birth/hatch to slaughter always operating at full capacity. Disruption of flow at any part of the chain will therefore have immediate impact both upstream and downstream, with likely immediate consequences for animal welfare but also for humans and the environment. The immediate impact of COVID-19 was a wave of panic buying by the public. Among the products to disappear from supermarket shelves in the first few days were toilet rolls, disinfectants and sanitizers, pasta, rice, flour, and yeast, and in some countries, eggs, cheese, and milk. General trends included increased meat, egg, and dairy retail sales with a sharp upward spike as lockdowns were announced (14), but then sustained sales when compared with year-on-year, from early March to July, where records are available (15). This was a consequence of the increase in meals being prepared at home, with schools, workplaces, and restaurants closed.

Countries such as the U.S. have two relatively distinct supply chains: one that supplies grocery stores and one that supplies the food service industry. Hence, gaps on shelves did not represent a shortage of commodity *per se* but the commodity existing in forms unsuitable for supermarkets compounded by distribution chains unable to cope with increased retail demand. As restaurants and schools closed, overall demand for dairy showed a 12–15% decline in the U.S. (16), leading to milk surplus and dumping. Whole egg demand increased but liquid egg demand, usually 30% of the U.S. egg market, decreased, leading to plant closures, contract cancellations, and the euthanasia of laying hens. The demand fell for high-end beef usually served in restaurants and farmers and processors struggled to cope with changing levels and types of demand from different sectors. However, the greatest impact of COVID-19 on the livestock product supply chain commenced with disease outbreaks among processing plant workers, leading to plant closures and effects up and down the food chain.

## COVID-19 Effects on Human Welfare

There are reports of clusters of COVID-19 cases in processing plants in several countries, including Canada, Brazil, U.S., Ireland, U.K., Spain, Australia, Denmark, and Germany (17). In Germany, coronavirus infected more than 1,500 workers in one of Europe's largest meat-processing plants (18). This represents a mass outbreak several weeks after the virus peaked and at a time when the country was “reopening.” However, the U.S. remains the hardest hit country where, according to one website, “As of September 11, there have been at least 39,000 reported positive cases tied to meatpacking facilities in at least 417 plants in 40 states, and at least 184 reported worker deaths in at least 50 plants in 27 states.” (19). Forty-nine plants were closed for various lengths of time (19), and nearly 200 U.S. Department of Agriculture—Food Safety Inspection Service inspectors tested positive, with four deaths (20). In a study of processing plants in 23 states, 9.1% of workers tested positive during April and May 2020 (21).

Apart from the obvious direct impact on human welfare for those who were infected and became ill or died from the disease, the clusters at processing plants highlighted several inequality issues that contributed to the outbreaks. Firstly, the vast majority of the workforce in meat plants represent migrant and minority workers who are inherently more vulnerable to exploitation (22) compounded by language barriers (23). There is evidence that the disease affects minority workers disproportionately, with Hispanic and Asian workers making up 30 and 6% of the workforce, yet 56 and 12% of the positive cases in U.S. plants (21). Additionally, the processing portion (including slaughter and packing) of farm-to-fork production is inherently more dangerous than non-food system industries (24). Meat, dairy, and fish production is more dangerous than other food production, with relatively high levels of severe equipment-related and assault-related injuries, and more fatalities from assaults from co-workers and animals and exposure to harmful substances (24), together with increased psychological distress among slaughterhouse workers (25). Those on the processing line work in very close proximity where food safety and the risk of zoonotic disease direct hygiene practices, rather than person-to-person disease spread. Superimposed upon these dangers, is evidence of low pay, lack of sick leave and affordable healthcare, together with high density and low quality housing for workers (26).

**Figure 1:**
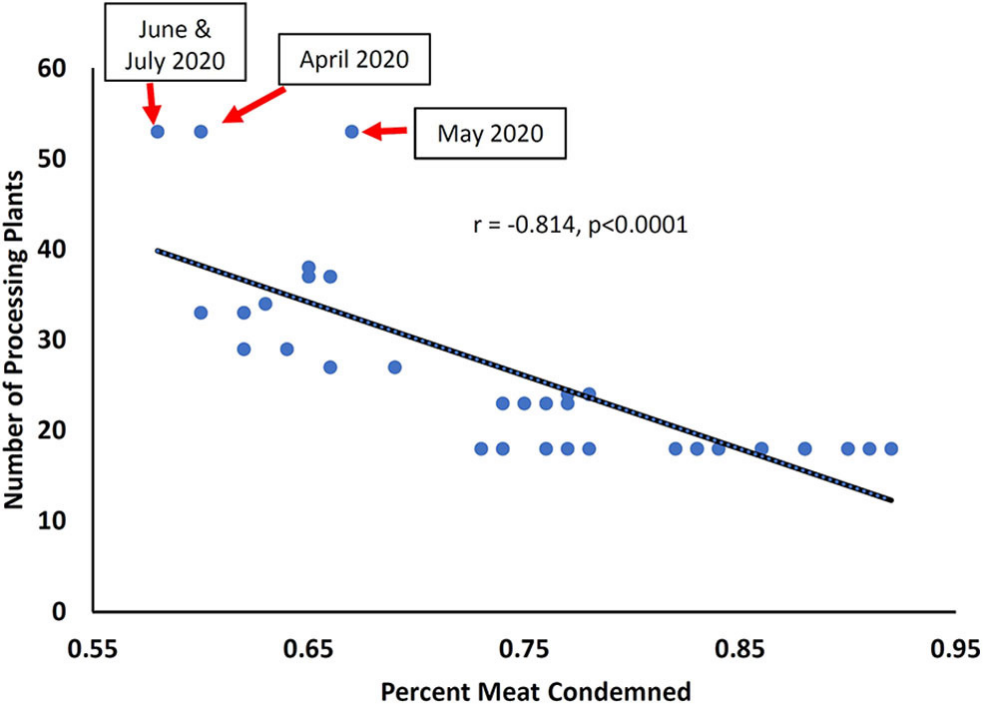
Relationship between number of poultry processing plants given line speed waivers by USDA-FSIS and the percent of chicken meat condemned by weight, between January 2017 and July 2020 [Sources: (27, 28)].

When processing plants started closing down, the affected workers faced financial uncertainty. Workers elsewhere in the supply chain also faced a period of insecurity when the effects of plant closures became apparent, including job losses, financial impacts, loss of animals, etc. Where plants were still working but with reduced staffing, workloads were increased or duties changed, both likely to increase risk of injury. In some instances, the limits on line speeds were raised by waivers from the USDA-FSIS, again likely increasing worker stress and injury risk, and with potential impacts on animal welfare (stunning effectiveness) and food safety. A record number of 16 poultry processing plants acquired line speed waivers in March and April 2020 (27). This allowed the number of birds being stunned and killed to increase from 140 birds/min to 175 birds/min. Faster line speeds likely contributed to the reduction in the post-mortem condemnation percent which fell 7.7% (a monthly record) to a record low of 0.60% condemned meat by weight (Figure 1) in April 2020. After a slight rebound in May, a new record low of 0.58% condemned meat by weight was set in June and July 2020. This indication of possible reduced inspection oversight is supported by the fact that between 2017 and 2020, there is a strong negative correlation between the number of plants with line speed waivers and percent of weight condemned (Figure 1). Ultimately, this represents a major threat to public health (and welfare) through reduced food safety (29).

Processing plant closures affected some farmers who, faced with nowhere to send animals for slaughter, had to prepare for and carry out mass depopulation of surplus animals. We detail the impact this had on animal welfare below, but mass depopulation also carries a human welfare cost, for the stockperson and for those tasked with carrying it out. Even at a single animal level, emotional strain on stockpersons is a barrier to the euthanasia of sick animals (30). When moving to a farm population level, the outbreaks of foot-and-mouth disease in the UK showed that affected farmers suffered increased stress, marginalization, and depression (31). The effect was more widespread within rural communities and included “distress, feelings of bereavement, fear of a new disaster, loss of trust in authority and systems of control, and the undermining of the value of local knowledge” (32). Those killing the animals are not immune to the impact, even without the emotional or financial ties of ownership/livelihood. Two years after the foot-and-mouth disease outbreak in Japan in 2010, veterinarians, livestock technicians and even clerical workers interacting with the farmers suffered mental stress (33). To that end, current guidelines include the recommendation that “to mitigate the negative psychological effects of involvement in mass euthanasia activities, psychological counselors should be made available to both staff and the stakeholders” (34).

Finally, a less obvious impact on human welfare is the public health risk posed by carcass disposal (35). Of all carcass disposal methods open air burning and unlined burial of carcasses pose the highest risks of contaminating ground and surface water, soil, and air with pollutants and pathogens like *E. coli* and *Salmonella* (34, 36). Though banned in many countries (36) the U.S. permits both methods for emergency disposal of carcasses (37). Composting is a frequently employed method of disposing of casualty animals on farm and it too poses similar risks if done at scale (37). Additional risks to public health are posed by vectors that feed on carcasses, such as birds, flies, and mosquitos as they can spread biological leachate components (38). On-farm burial and composting, “in-house” in the case of poultry, were among measures employed to dispose of carcasses in the current pandemic. Concerns were raised for public health in areas where carcasses were disposed of using such methods not only because of the risk of pathogen spread but also because of odor and flies (39, 40). Additionally, USDA-APHIS (34) acknowledge the potential for psychological harm caused by the “extremely unpleasant odors and sight of animal remains.” Inhabitants of areas where carcasses were disposed of at scale may already be disadvantaged in terms of their health and welfare (41). The air around pig and poultry sites contains hydrogen sulfide and ammonia, particulate matter, and bacteria (42). Such pollutants act as eye and respiratory irritants (43). Unsurprisingly then, inhabitants are more likely to suffer more from asthma and other respiratory diseases (44). Exposure to these pollutants also contributes to mental stress (45) and elevated blood pressure (46). Hence, the threats to public health associated with carcass disposal may compound existing health challenges for people in the surrounding population and may even place them at higher risk of serious complications or death should they contract COVID-19 (40).

## COVID-19 Effects on Animal Welfare

**Figure 2:**
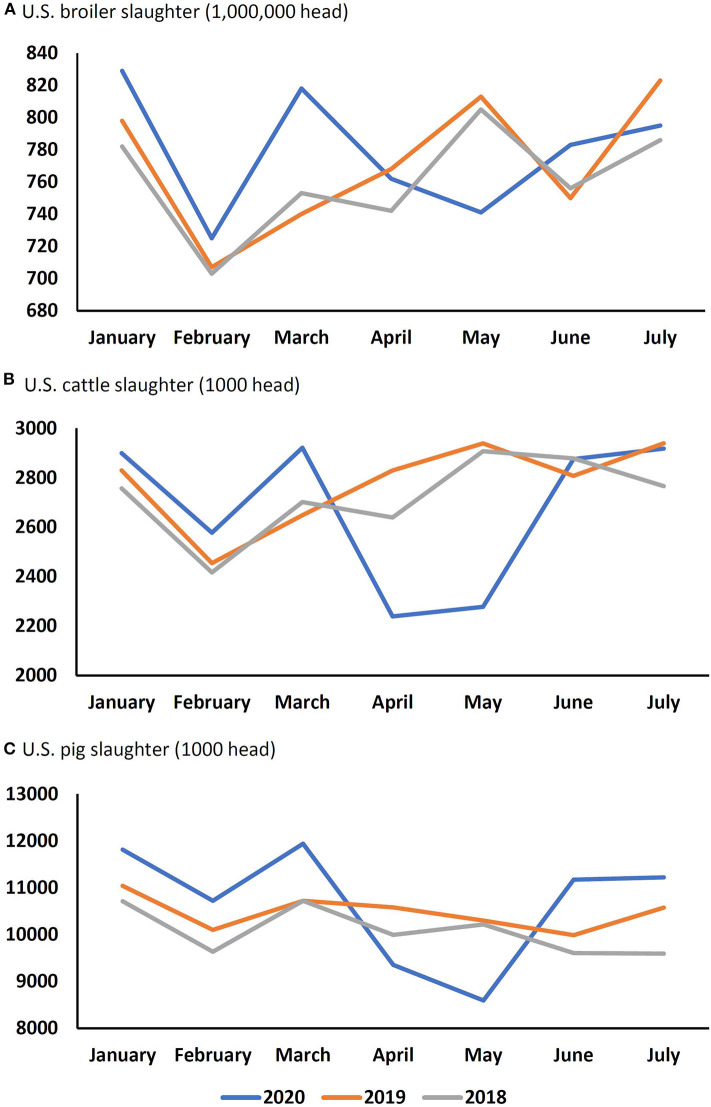
Numbers of **(A)** broiler chickens, **(B)** cattle, and **(C)** pigs slaughtered per month in the United States between January and July over the last 3 years [Sources: (28, 49)].

The biosecurity and pollution risks posed by mass carcass disposal outlined in the preceding section could also adversely affect the welfare of wild animals, fish, birds, and insects which are not discussed in the current paper. Here we focus on the effects that human clusters of COVID-19 at meat processing plants and the associated decisions to close them, had on animal welfare. Within the U.S., the closures began with a Foster Farms poultry processing plant at Farmerville, Louisiana on March 27th 2020 (19). Over the next 4 weeks, a cascade of closures across cattle, poultry, and pig sectors followed—some closures were only for a few days for deep cleaning, others were longer (47). The result was a loss in slaughter and processing capacity. By the 4th week in April, it was estimated that pig slaughter capacity in the U.S. was operating at only about 55% of normal (48), meaning that about 250,000 pigs a day were at slaughter weight, but had nowhere to go for slaughter. The impact was similar for other livestock industries reflected in the monthly data for all species (Figure 2).

By April 28th 2020, the U.S. President invoked the Defense Production Act of 1950, and issued an Executive Order mandating processing plants to reopen. Since then, plants reopened, but many with reduced capacity due to staff shortages. By May 19th, pig slaughter capacity was back to 79.3% of normal, but this still represented a shortfall of over 100,000 pigs per day. For poultry, increasing the number of processing plants operating with line speed waivers recaptured some of the reduced capacity. This allows the number of birds being stunned and killed to increase from 140 birds/min to 175 birds/min. This possibly increased the number of birds exposed to incomplete stunning which poses major concerns for animal welfare (50). With the slaughter end of the chain experiencing reduced capacity, there is an almost immediate impact on animal welfare on farm mostly arising from overcrowding. As detailed above, the poultry and pig industries in particular are intensive and integrated, with little or no flexibility within the production system. When pigs and chickens are unable to leave the farm for the usual slaughterhouse at the designated time, there is an immediate “bottleneck” in the system, because the “production” of new chicks and piglets continues unchecked. With longer gestations and slower growth rates, cattle production is under less immediate pressure. In some cases, there is extra slaughter capacity at other processing plants, but this may increase transportation time and distance, exposing animals and birds to increased transport stress (51).

Intensive pig and poultry production systems are characterized by maximal use of buildings, maximizing the number of chickens or pigs per square area, and the number of days the pens or buildings are in use per year. Each farm has a pre-determined flow with rigid set dates for the animals to enter and leave, based on expected growth rates. Broiler chickens arrive on farm as day-old chicks and are ready to leave at slaughter weight 6–7 weeks later. With a 3-week egg incubation period, the whole production cycle is 9–10 weeks. Pigs in the U.S. move through the farrowing house (3 weeks), the nursery (6–8 weeks) and the grow-finish barn (16–17 weeks) before slaughter. With a nearly 4-month gestation period, the pig production cycle is 41–44 weeks. Without the ability to move livestock off the farm, serious overcrowding occurs within days or a few weeks at most.

For broiler chickens, their phenomenal growth rate causes almost immediate problems in terms of lack of space. For example, if stocked at the maximal EU stocking density of 33 kg/m^2^ under minimum welfare standards, this equates to about 13 birds/m^2^ at 6 weeks of age (about 2.5 kg/bird). By week 7, there is 42 kg/m^2^ and by week 8 there is 48 kg/m^2^. EU farms that meet certain extra requirements can stock up to 42 kg/m^2^ (52) and meeting this target at 6 weeks of age equates to about 17 birds/m^2^. By week 7, there is 54 kg/m^2^ and by week 8 there is 62 kg/m^2^. Hence, overcrowding from a legal definition occurs within 1–7 days. From a welfare perspective, high stocking densities can lead to decreased walking ability, poorer leg health, increased fearfulness, increased footpad and hock dermatitis and increased mortality (53). Overcrowding-induced increased heat production and associated reduced environmental qualities, such as poorer air and bedding quality exacerbates these welfare issues.

Pigs are selected for increased growth rates but the fact that the birth-to-slaughter time period is 24–28 weeks and is a multi-stage process, means the industry is slightly more flexible compared to broiler production. Modeling exercises determine the impacts that imposed movement restrictions may have with respect to an outbreak of a foreign animal disease (FAD), such as African swine fever (ASF). Increasingly, pig production is on multiple sites, with piglets moving off the breeding farm at weaning or after nursery phase. Modeling for FAD assumes no movement between units whereas the COVID-19 situation allowed it. Without movement between units, breeding-only units can reach critical overcrowding in 4–5 days. Nursery units take 24–52 days, grow-finish units take 78 days and farrow-to-finish units take 43 days (54, 55) to achieve crowding. Effects of crowding for pigs includes decreased general activity and comfort behaviors, increased aggression, skin lesions and tail injuries, increased foot and limb injuries, reduced growth and physiological function, and increased susceptibility to disease (56). The latter increases use of antimicrobials, which in turn increases the risk of antimicrobial resistance.

Clearly, fast growth rates are a major factor in overcrowding. In pigs, methods to decrease growth rates include removal of growth promoters, moving to lower energy diets, reducing feed availability and increasing building temperature to reduce appetite and hence, feed intake (57). Removing growth promotors is likely to improve pig welfare (58). However, anything that reduces feed intake may lead to animals experiencing hunger, a negative affective state (59) and reduced satiety may also lead to increased aggression as animals seek to gain access to a limited resource (60). Likewise, inducing heat stress has detrimental effects on pig welfare (61).

**Figure 3:**
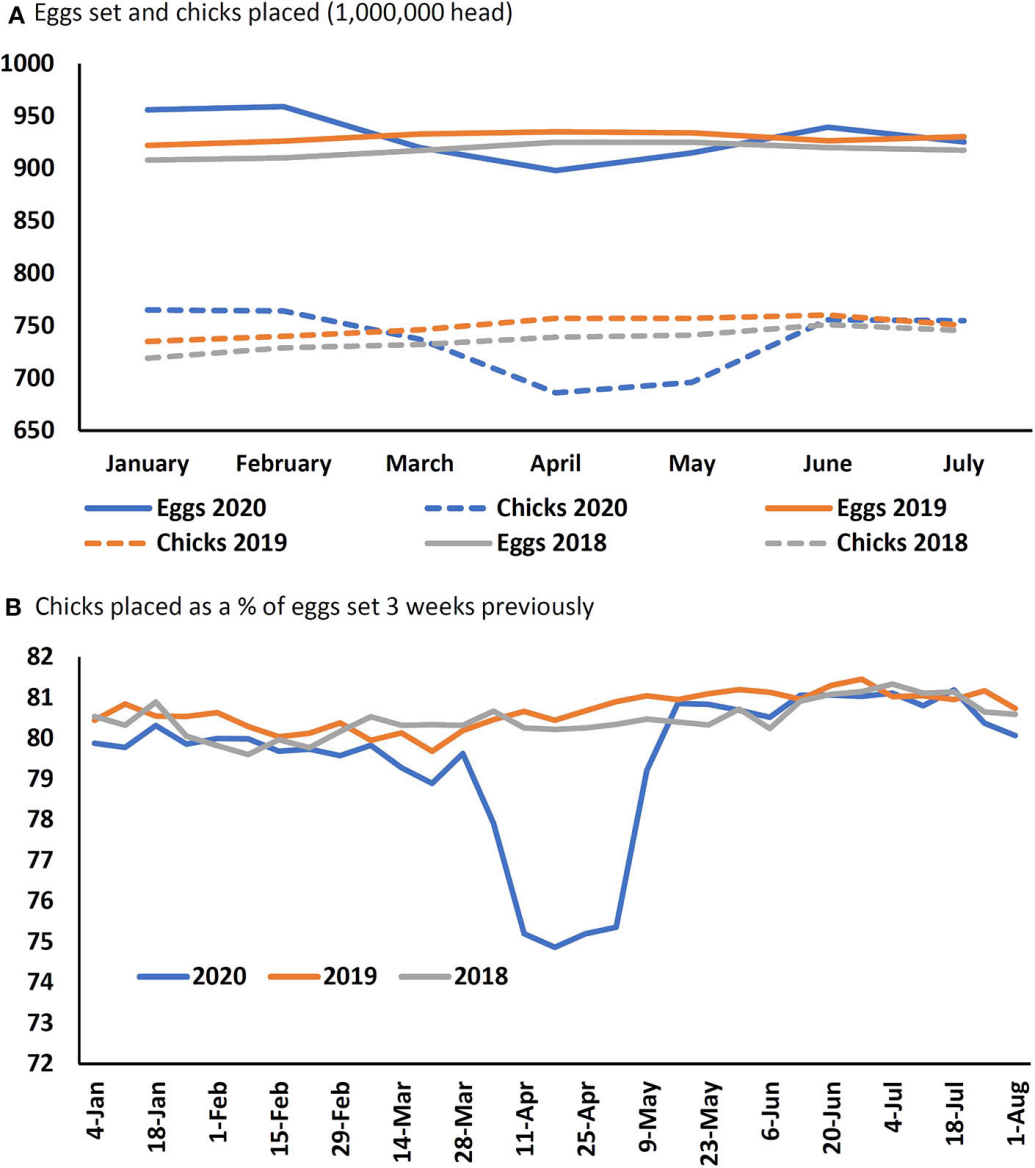
**(A)** Numbers of eggs set and broiler chicks placed between January and July, and **(B)** broiler chicks placed as a percent of eggs set 3 weeks previously between January and July over the last 3 years in the United States [Source: (68)].

One way to slow or stop new animals and birds entering at the input end is to stop breeding the females. However, as the gestation length is nearly 16 weeks in pigs, it would take that long to feel the impact of this measure. Inducing abortions would have an immediate effect in terms of easing space in the farrowing house, which could be repurposed as nursery pig accommodation. This would relieve pressure up the chain, but only on a temporary basis. For poultry, the chain is much shorter, and a reduction in eggs entering the incubator, results in a reduction in bird numbers within 3 weeks. Alternatively, eggs can be removed from the incubator and euthanized, or chicks killed at hatching. The recommended methods for egg euthanasia are dependent on the stage of incubation. The American Veterinary Medical Association (AVMA) Depopulation Guidelines (62) recommend that eggs that are >80% incubated (day 16, chickens; day 22, turkeys, ducks) be treated as per newly hatched chicks, and subject to preferred methods that “include containerized gassing, cooling, freezing, and maceration.” Eggs <80% incubated can be euthanized by freezing, cooling to <4°C for 4 h or exposure to high CO_2_ concentrations for at least 20 min. Implications for animal welfare of euthanizing eggs are unknown but there are considerable welfare, ethical, and societal concerns surrounding the killing of day old chicks (63, 64). Maceration is often used for chicks up to 72 h old, and under EU regulations, maceration “should result in instantaneous maceration and death of the chicks and embryos (unhatched eggs). The apparatus should contain rapidly rotating, mechanically operating blades.” There are a number of identified hazards that may prevent this from happening, such as slow equipment and overloading by handlers (65) and there is likely an increased risk of such hazards when both machines and workers experience higher than normal throughput. Maceration is banned in Switzerland, France, and Germany. Gassing also carries welfare concerns (66, 67), especially with CO_2_, with one study concluding that “behavioral signs of distress were observed with all treatments, and occurred at concentrations lower than those causing insensibility” (67). There is some evidence that the U.S. broiler industry carried out egg and/or chick euthanasia, with marked reductions in eggs set and chicks placed, and a lower percentage of set eggs being placed (Figure 3).

The worst-case scenario is where the only resolution to the backlog of animals is to kill them on farm. Ideally, this would be by euthanasia, whereby animals have a “good death” without pain or distress. At the very least, emergency killings should observe the same level of animal welfare as during planned killings or standard slaughter. This means as little handling as possible and use of a killing method that either causes immediate death, or sedation followed by death, or death in already stunned/unconscious animals (69). However, this is difficult to achieve when killing animals at scale in an emergency. The most recent widespread need for mass depopulation of animals was in the control of ASF outbreaks and disturbing videos emerged of the burial and burning of live animals in Asia. Within the U.S., the AVMA released updated guidelines in 2019 (62), for use in conjunction with FAD PReP/NAHEMS Guidelines: Mass Depopulation & Euthanasia (34). The Guidelines detail appropriate methods by species, in terms of “Preferred,” “Permitted in Constrained Circumstances,” and “Not Recommended.”

For pigs, the “preferred” methods include gunshot, non-penetrating captive bolt, penetrating captive bolt, electrocution, manual blunt force trauma, carbon dioxide (CO_2_) and anesthetic overdose, though the applicability of each method is also dependent on size and age of the pig (62). Permitted in constrained circumstances are ventilation shutdown in combination with additional heat or CO_2_ (abbreviated as “VSD Plus”), and dosing with sodium nitrite (62). However, there is little research on some of these methods. For example, sodium nitrite was previously only used in the control of feral pig populations (70) and never for mass depopulation of commercial pigs. Its efficacy is contingent upon pigs being able to ingest a toxic dose in a limited and acceptable, non-defined timeframe (62). As the COVID-19 crisis emerged, the U.S. National Pork Board issued an emergency request for proposals entitled “Animal well-being depopulation field trials” with a deadline of May 11th 2020 to identify projects and started by May 29th 2020. This highlights the paucity of information for pigs. The exact numbers of healthy pigs killed as a consequence of COVID-19 is not yet available, but officials in Iowa, the top pig-producing state in the U.S., estimate that 600,000 animals may need to be euthanized in the state alone (71).

For poultry, different methods are approved depending on whether the birds are indoors or outdoors and if they are floor-reared or caged (62). For floor-reared birds, such as broilers or aviary-housed laying hens, “Preferred methods include water-based foam generators, water-based foam nozzles, whole-house gassing, partial-house gassing, containerized gassing, cervical dislocation, mechanically assisted cervical dislocation, and captive bolt gun. Methods permitted in constrained circumstances include gunshot, VSD plus, controlled demolition, exsanguination, and decapitation” (62). For caged birds, “Preferred methods include whole-house gassing, partial-house gassing, and containerized gassing. Methods permitted in constrained circumstances include compressed air foam, cervical dislocation, mechanically assisted cervical dislocation, captive bolt gun, VSD plus, and decapitation” (62). Whole-house gassing using CO_2_ emerged as the major method of choice, together with water-based foam methods. Importantly, The World Organization for Animal Health (OIE) does not condone water-based foam for euthanasia, even in situations of emergency disease control (72). Recently, the European Commission tasked the European Food Standards Agency to examine the scientific evidence surrounding mass euthanasia of farm animal species and identify hazards to animal welfare. The report concerning poultry identified 29 potential hazards, of which 26 were associated with the personnel carrying out the task (65). For both whole-house gassing and foam methods, insufficient time of exposure was a hazard. Timing of the accompanying VSD needs to be appropriate so that the chosen method is the cause of killing, rather than thermal stress caused by VSD itself. As with pigs, the exact number of poultry euthanized due to COVID-19 is currently unknown, but there are reports of the culling of up to 10 million chickens in the U.S. (73).

The potential negative impact of mass depopulation on the welfare of animals and birds is likely enormous. At its most extreme, distressing videos emerged of the need for additional captive bolt killing of pigs still alive after “2–3 h of 140°F heat” following use of VSD Plus (74). Correspondingly, World Animal Protection called on the AVMA to remove this and water based foams from its guidelines of currently approved methods for the depopulation of animals as it causes prolonged heat stress and suffocation (75). In fact there are three major factors influencing animal welfare during the depopulation process (69): (i) handling prior to killing, (ii) the stun/kill quality, and (iii) confirmation of death prior to carcass disposal. Most methods of killing have limitations in one or more of these factors (62). For example, there may be a trade-off between possible distress during a longer time to induce unconsciousness and the benefits of reduced handling of individual animals associated with a particular method. The subjective feelings of the animals subjected to mass depopulation are likely to include, fear, pain, and distress potentially reflected in open-mouth breathing, ataxia, righting responses, escape attempts, and vocalizations (76) among other behavioral signs of suffering.

## COVID-19 Effects on Environmental Welfare

Even under normal circumstances, carcass disposal methods pose a pollution risk (35). However, there are major environmental implications associated with disposing of carcasses at scale (38, 77). Furthermore, as pig and poultry industries are often concentrated in specific geographical areas, killing thousands of animals and birds may create a new stream of waste in ecosystems already burdened by environmental pollution [e.g., (78)]. Generally, as carcasses degrade, bodily fluids, chemical and biological leachate components and hazardous gases [e.g., ammonia (NH_3_), hydrogen sulfide (H_2_S), methane (CH_4_), and other air pollutants] are released into the environment, including into the air, surface water, and groundwater. The extent to which there is a risk of this occurring obviously depends on the chosen method (38). However, some of the more risky methods of carcass disposal (38) were employed in the U.S. including unlined burial and composting (40, 79). Both are prohibited in many countries including The European Union (E.U.) under the EU Animal By-Product Regulations 2014.

Composting is a carcass disposal method that promotes decomposition through placement of carcasses between layers (approximately two feet thick) of carbon rich organic materials. With the need for mass carcass disposal, massive quantities of materials like wood chips, corn stalks, sawdust, or straw were needed placing a drain on environmental resources. Under normal circumstances, composting has potential to contaminate the underlying soil and is associated with greenhouse gas (GHG) emissions (35). At scale, increases in ammonia-nitrogen appear to pose the most significant soil pollution hazard (80). Such risks are minimized by use of an impervious base layer, regular turning and covering the compost heaps (37) but this is difficult to achieve in an emergency. Some poultry producers composted chickens in the houses where they were killed by layering the carcasses with straw and “cooking” them under high heat for about a month in what is likely an energy intensive process. However, it can be difficult to successfully compost carcasses in non-purpose built or other “make-shift” type compost facilities resulting in increased GHG emissions (39). Furthermore, dead chicken compost is spread on fields similar to fertilizer and such land application poses “run-off” concerns as it is even higher in phosphorus than manure (81).

At the time of writing, there are no reports of mass depopulation in the E.U. but the need for carcass disposal at scale could change with continuing closures of meat processing plants. If required, it would likely have to be by incineration (either on or off-farm) and rendering both of which are less risky to the environment in terms of contamination (38). However, it is widely acknowledged that neither process could cope with carcass disposal at scale. Assuming limitations with capacity in the few remaining rendering plants operational in the E.U. (35) could be overcome, the process still has a high-energy demand and produces effluent with high biological and chemical oxygen demand. Net GHG emissions can be minimized if some of the by-products (e.g., tallow) are recovered for subsequent energy production. The process also means animals are not wasted completely as rendering claims to recycle meat, bone, and fat into ingredients for numerous products. Incineration of carcasses is also highly energy intensive, exacerbated by the relatively high water content of carcasses meaning that it generates considerable GHG emissions. Large-scale mobile waste incinerator units could be used to process massive volumes of animal carcasses in a biologically safe way. However, there are issues with the operation costs and turnaround time, and ash disposal may cause environmental challenges. There are more environmentally friendly methods of carcass disposal (38). However, processes such as alkaline hydrolysis, are currently too expensive for use in anything but highly specialized operations (82).

As mentioned earlier, killing of poultry in the current crisis often involved foam methods. Water-based compressed air foam (CAF) has its origins in firefighting; CAFs reduce the total water supply to extinguish a fire to as little as one-third compared with applying water alone. However, as a form of mass euthanasia they use copious amounts of water (37). They also contain chemical surfactants and preservatives and certain biological nutrients. Hence, in the case of protein-based foams, breakdown in the environment releases ammonia. Other reported environmental concerns include water pollution/de-oxygenation and the accumulation of the associated compounds in plants and animals (83) although the extent to which these are potential problems associated with its use in mass depopulation of poultry is unknown.

In a somewhat related environmental problem, numerous countries dumped hundreds of thousands of liters of milk because of the fall in demand [e.g., (84)]. Milk dumping poses a serious risk to fish and aquatic life as it reduces oxygen levels if it gets into waterways due to its high biochemical oxygen demand (85). In the U.S., farmers were advised to hold milk in manure storage lagoons if high rainfall was expected as this causes even faster runoff. However, such manure storage lagoons are themselves prone to failure during particularly high rainfall events (86).

Some dairy cooperatives advised farmers to cull extra cows (87). Any form of involuntary culling of animals raises GHG emissions (88). Hence, emergency disposal of farm animals (and their products) represents a dramatic increase in the carbon footprint of food production systems. At its most basic, it also represents an enormous waste of the finite resources (land/feed, water, and fossil fuels) that went into producing those animals and birds in the first place.

## What Can We Do to Lessen the Impact?

The post-World War II industrialization of agriculture was successful in its immediate goal of increasing the amount and affordability of food in developed countries. The costs associated with such a sustained push for more plentiful, cheaper food generally remained hidden when the system was functioning, supported by the general dissociation of food production from food consumption (89). Not surprisingly then, perturbations about our models of food production were mainly related to direct and immediate threats to human health caused by food safety emergencies such as “mad cow disease” or dioxin scares (90, 91). In recent years, the number of publications relating to the additional threats of climate change, biodiversity loss, and antimicrobial resistance associated with food animal production increased (92–94). However, the COVID-19 pandemic revealed the harsh reality about the fragility and high “costs” associated with intensive and highly specialized food production systems like no other threat before (95). The unequivocal detrimental and interrelated implications of COVID-19, for humans, farm animals and the environment outlined in this paper, provides compelling evidence of complex interconnectivities that are captured in the One Welfare concept (12). Indeed, One Welfare reconnects food production with food consumption, the diametric ends of the production chain. Apprehending this connection is crucial if we are to undertake the radical overhaul required of the way in which we currently raise, kill, process, market and consume meat, and dairy products (96, 97). With many environmental systems and processes being pushed beyond safe boundaries by food production the need for change is urgent (97). Indeed, there are threats that a new pandemic is imminent at the time of writing (98). Changes are required at all stages from production through processing and retail to consumption (99, 100). In the ensuing sections, we initially suggest some of the immediate, more short-term solutions that could be implemented at each of these stages.

### Production

The need for emergency killing of animals and birds at scale on farm as well as the strategies employed to reduce throughput (slowing growth rates, induced abortions etc.) pose major problems for One Welfare as outlined above. Therefore, in the face of more frequent threats similar to the current COVID-19 crisis, it is imperative that such strategies are avoided completely. Achieving this likely requires the transformative change of current animal production systems referred to above so there are not many immediate, short term solutions. Indeed the tentative suggestions below come at a cost to some aspect of One Welfare and should be limited to exceptional circumstances.

A relaxation of quality assurance/premium product standards, i.e., Global Animal Partnership 5-step Animal Welfare Standards (101), RSPCA Assured (102) etc., without penalty would benefit farmers' emotional welfare as it would prevent depopulation based on failing standards. For example, RSPCA Assured space allowances for 50–85 kg pigs are 0.55–0.675 m^2^ per pig. Global Animal Partnership's space allowances for 50 kg+ pigs is 0.93 m^2^ on Step 1 or 1.10 m^2^ on Step 2. E.U. minimum space allowances for 50–85 kg pigs is 0.55 m^2^. If pigs are unable to leave the farm for regular slaughter due to processing plant closures, pressure on space within the system increases. Without relaxation of standards, farmers would have to euthanize animals or risk losing premium payments for failing to maintain scheme standards. Hence, temporary relaxation could not only reduce the number of animals depopulated, but also reduce stresses associated with impending financial distress for the farmer. Clearly such standards assure better quality of production methods with associated benefits to animal welfare and food safety so other aspects of One Welfare would suffer.

The U.S. National Pork Board suggested moving animals into temporary housing or outdoors (103) as a temporary solution to the problem of surplus animals. However, this was an unrealistic option for many producers, for obvious reasons such as lack of an additional, suitable spaces. This “solution” could also constitute an animal welfare risk due to potential exposure of animals to adverse weather, inappropriate climatic conditions (for weaned pigs for example) and difficulty supplying feed or water outdoors. Nevertheless, there is undoubtedly merit in documenting available/empty buildings, land, or other areas that might be suitable to accommodate surplus animals. Producer groups or large integrators could co-ordinate these databases at national or local level and across species. Such a solution would also be useful to help in moving animals to safer locations if there was threat of natural disasters such as flooding. However, it would not be useful in the face of an infectious threat to animal health where movement is prohibited (as in the case of ASF).

### Processing

Ideally surplus/additional animals would be slaughtered in the usual way (69) but currently this is not possible given the reduction in processing plant capacity across all species (Figure 2), and in many countries worldwide (17). The June 17th estimate for the U.S. pig industry, was a backlog of 3.2 million pigs (104). As with the trend in farm numbers and sizes, there is a similar trend in processing plants, with fewer plants processing larger numbers of animals. In 1970, there were over 7,000 processing plants. In 2020, there are about 2,700, of which the U.S. federal government inspects 835 and which account for about 99% of total slaughter capacity. The range in capacity within these 835 plants is large. For cattle, the U.S. federal government inspects 670 plants but the 12 largest slaughter 52% of the total number. For pigs, the U.S. federal government inspects 619 plants and the 14 largest slaughter 59% of the total number. Hence, if the largest processing plants are closed, the smaller plants have insufficient spare capacity to make up the shortfall, and so until the point at which the processing plants are back online, short-term remedial action could include the following options. Increase capacity at open processing plants. Plants remaining open can achieve short-term increases in capacity by increasing hours of operation, and relocating healthy staff from closed plants. According to the National Pork Board, pig processing plant capacity is based on the plants being open, on average, for 5.4 days per week (48). If individual plants could stay open for 7 days per week, they could increase their capacity by nearly 30%.

There are a myriad of reasons for the worldwide decline in the number of abattoirs, however, burgeoning food safety regulations represent a significant financial burden for these small businesses (105, 106). Such rules are increasingly aligned with global standards and therefore developed for the intensive, large-scale food system, which makes them antagonistic to the practices of small-scale farmers, and local production systems (107). Efforts to address the decline in local abattoirs should include a broadening of the scope of risk analysis (108) to incorporate the benefits to One Welfare associated with local slaughter in small or mobile abattoirs (109, 110).

In the immediate term, it is clear that we need to protect the welfare of humans working in the processing sector better. The meat processing industry is a difficult working environment and regardless of country, there appears to be an increasing reliance on migrant labor to fill positions in what is known as a high employee turnover industry. For example, countries in Western Europe have many migrant workers from Eastern Europe (111). The United States has many migrant workers from Latin America (112). Many plants do not have unionized workforces and many employ undocumented or sub-contracted workers on low wages, with the tacit acknowledgment that the power and major economic benefit lies with the employer. The combination of these social factors and physical factors within the workplace (proximity, ventilation, aerosolization) made processing plants ideal hotspots for clusters to emerge (113). The Centers for Disease Control and Prevention and the Occupational Safety and Health Administration issued joint guidance for processing plants in the U.S. after closing and cleansing (114). Some of these measures include temporary modification of the physical environment; especially increasing spacing between workers, but the longer-term solution requires redesign of processing plants (113). Other guidance focused on the workers, including guidelines to isolate from others during travel to and from work, and staying away from work and seeking medical attention when sick (114). This advice is well meaning, but the current reality is that the combination of low pay, lack of sick leave and medical insurance, crowded social housing and lack of public transport means that much of the advice cannot be followed. There will need to be a longer-term commitment from the processors themselves to invest in their workforce, and improve work conditions, pay, and access to healthcare. The German Agriculture Minister has been vocal in decrying that meat has become a cheap product which does not equate with sustainability, and will introduce legislation to force processing plants to hire employees directly, to end the sub-contract culture and improve worker pay and conditions (115). This will incur cost, and that cost should be met by the combined actors up the supply chain, including retailers and consumers.

### Retail

Vertical integration and reliance on large, centralized meat processing plants means there are many opportunities for bottlenecks in the long chain between farm and retail when challenges arise. Distinct differences in the U.S. between supply chains for grocery stores and the food service industry exacerbates this and contributes to the inflexibility of the system. Aligned with a shift amongst certain consumers to the practice of “consumption for the greater good” demand increased for “local” produce or “slow food” in the last few decades (116, 117). This is supplied either through farmers markets (118), or through Community Supported Agriculture. In the latter, families buy “shares” in a farm which supplies them mostly with fruit and vegetables, but also meat, eggs, and dairy, throughout the year (119). Uptake increased greatly during the current pandemic as did interest in these “direct-to-consumer” retail models with some having waiting lists in the hundreds (120). Closure of meat processing plants prompted some farmers to explore alternative methods of sales and distribution. This involved use of online platforms and direct marketing (121), together with farmers markets (122), where still open, and partnering with dine-in restaurants, moving to home delivery. However, such alternative methods are more accessible to those producers who are already part of shorter supply chains, such as those in niche markets or certification schemes such as organic and high welfare (123). Other advantages of this direct approach is economic gain for the farmer and affordability of high quality food for the consumer. For example, organic food in Brazil can be sold direct at farmers markets, without the sometimes 400 times mark-up seen in supermarkets (124).

### Consumption

There were several immediate effects of COVID-19 on consumption patterns which if sustained could improve One Welfare. Working from home and the closure of schools and restaurants increased consumption of meals at home, shifting purchasing patterns from restaurants to supermarkets (125). While supermarket freezers were initially emptied of pre-prepared meals there was also increased purchasing of basic ingredients, highlighting an increase in in-home preparation of meals (126) which has potential benefits to human health (127). Sales also soared for meal kit companies, such as Blue Apron and HelloFresh, and also for plant-based meats such as Impossible Foods and Beyond Meat, which saw a 264% increase in sales over March–May 2020 (128). This increase in consumption of plant-based alternatives to meat is controversial. These popular brands are highly processed, and they may be of doubtful benefit to human health, often being served as a meat substitute in an otherwise unchanged “fast food” diet (129). They may have benefits for the environment in that “A Beyond Burger generates 90% less GHG emissions, requires 46% less energy, has >99% less impact on water scarcity and 93% less impact on land use than a ¼ pound of U.S. beef (130).” but these products do contain ingredients from monoculture agriculture and should still be sourced with ethical responsibility. Highly processed plant-based meat alternatives may have a role to reduce overall meat consumption—a 50% reduction of which would have an estimated 35% reduction in GHG gases, a 51% reduction in food's land use (131)—but a diet rich in unprocessed plant-based foods will be more beneficial to One Welfare. A shift away from Western-style, high meat-based diets to others such as the Mediterranean Dietary Pyramid (132), would impact human, animal and environmental welfare, increasing the sustainability of food production and consumption (133).

## Long Term System Overhaul

COVID-19 revealed that our current, large-scale, vertically-integrated food systems lack resilience or the capacity to adapt over the short term in the face of disturbance. A proposed food system resilience action cycle (134), would see a system encountering a shock (such as COVID-19 pandemic), absorbing it, reacting to it, restoring output to pre-shock levels, but also learning and building robustness ready for the next disturbance, so that its effect is dampened. We identified some potential learning moments and suggested changes to different components of the food chain to protect One Welfare in the face of future pandemics such as COVID-19. However, the process of building such resilience into the food chain will likely protect One Welfare irrespective of whether the challenge is related to another pandemic or to the “the elephant in the room”—climate change. Climate change represents the biggest threat “with the most unknown consequences for agricultural sustainability” (135), with adverse weather events, drought, flood, and wildfire events becoming more frequent (136) and livestock and crop pests extending their geographical reach (137). Ensuring food system resilience in the face of grand challenges can only come from a global transformation of the current model given that much of the world's population is inadequately nourished and many environmental systems and processes are pushed beyond safe boundaries by food production (97). We are perhaps fortunate that the COVID-19 pandemic has elucidated what lies ahead and, if acted upon, will enable us to hit reset and change while we still can.

Globally, we need to move away from the concept that Western industrialized agriculture, and aquaculture, and especially its intensive livestock and fish production systems, are the panaceas that will end food insecurity, even with a growing population and the increasing demand for animal protein. For global animal agriculture, at any one time, there are about 25 billion poultry, 2.25 billion sheep and goats, 1.5 billion cattle and 1 billion pigs (138). Every year, we consume 50 billion chickens, 1.5 billion pigs, 1 billion sheep and goats and 300 million cattle and about 173 million tons of fish, with about 80 million tons farmed (139). These numbers are currently increasing year-on-year as the industrialized model of livestock and fish production spreads to developing countries, especially in Asia (140) with associated increases in antimicrobial use (141) and other costs to One Welfare. The damage associated with the overriding focus on production efficiency could be addressed by a more holistic interpretation of efficiency such as that offered by the One Welfare approach which safeguards animal, human, and environmental welfare.

The European Union Farm to Fork (F2F) Strategy (142) appears to have a One Welfare approach at its core with its aim to transform food production into a “fair, healthy and environmentally-friendly food system.” F2F proposes changes to the whole supply chain, focusing on sustainability at all stages—shortening the chain and moving away from the “industry to fork” system (143), and adopting methods to reduce the environmental impact of production, manufacture, processing, retailing, packaging, and transportation, while preserving affordability, ensuring fair distribution of economic returns and safeguarding agri-food workers' safety and welfare. The strategy also states that animal welfare must be improved and that there must be less reliance on pesticides and antimicrobials, and biodiversity loss reduced and reversed. F2F acknowledges the interconnectedness of the planet and the global nature of trade, and hence that “change” needs a global approach. As the EU is the world's major exporter and importer of food it is well-positioned to influence global transformative change by adapting trade policies aligned to the One Welfare approach.

Likewise, the United Nations Sustainable Development Goals (SDGs) will help guide transformation of our food systems (144). One Welfare is implicitly embraced in the 17 goals to “end hunger, achieve food security and improved nutrition and promote sustainable agriculture.” For example sub-Goal 2.4 states that “By 2030, ensure sustainable food production systems and implement resilient agricultural practices that increase productivity and production, that help maintain ecosystems, that strengthen capacity for adaptation to climate change, extreme weather, drought, flooding, and other disasters and that progressively improve land and soil quality” (144). At first glance, animal welfare only applies to Goal 2 though even here it is not explicitly mentioned. However, a deeper examination of the SDG agenda revealed that out of 169 targets, 66 are relevant to animal welfare (145). More importantly, relationships between the SDGs and animal welfare were all positive, such that there was no situation where attainment of the SDG conflicted with improving or safeguarding animal welfare (145).

In spite of the tacit One Welfare approach in the F2F strategy and the SDGs, the lack of a specific focus on animal welfare is concerning. As animal welfare scientists, we are convinced of the importance of animal welfare in the development and delivery of solutions to global challenges while urging engagement as part of the interdisciplinary teams working on them (7, 146). In fact, framing animal welfare as the primary driver within the One Welfare concept is likely well-founded for a number of reasons. Firstly, there is a strong relationship between caring for animals, for a species, and for an ecosystem and this relationship is key to encouraging humans to conserve resources and protect the environment [e.g., (147)]. Farm animal welfare also plays a major role in driving animal health, performance and food safety all of which are crucial to the sustainability of animal production systems (148–150). In fact, improvements to animal health leads to a similar reduction in the carbon footprint of livestock farming as breeding for higher productivity but without the associated costs to welfare (151). We urge the strong support of such “win–win” strategies as they address both environmental and ethical sustainability. We stress that the consequences of current and future strategies for animal welfare must be scrutinized and contrasted against their effectiveness in mitigating climate change to identify the most cost-effective measures for improving environmental sustainability of livestock production. Similarly, others conclude that the welfare of farmed and wild animals should be central to the development of sustainable agriculture (152). This is even though concerns have been voiced (and allayed) that increased agricultural efficiency will inevitably conflict with animal welfare (148).

Hence, intensification *per se* is not necessarily bad, but it is imperative to practice sustainable intensification (153). Indeed, notwithstanding the problems posed by the focus on production efficiency, there is a need to increase agricultural output globally to deliver sustainable food security. However, there must be simultaneous progress on inputs such as moderating demand for livestock products (100) and decreasing food wastage, estimated at between 11 and 60% depending on the commodity (154). There should be focus on increasing yield per unit of current cultivated land mass, rather than increasing quantity of cultivated land mass. It may be that yield increases cannot be achieved everywhere and that some land is appropriate for management systems that promote biodiversity whereas other land may not be, and that it is better to intensify and “sacrifice” some land to monoculture agriculture, leaving other land to maintain full biodiversity rather than impact the biodiversity of all land to some extent. Others would argue that such a “land sparing” approach assumes that the functionality of biodiversity in agroecosystems is negligible (155). They suggest that a “land sharing” approach acknowledges the crucial ecosystem services provided by wildlife friendly farming and agroecological intensification. Silvopastoral systems, pastures with shrubs and trees as well as herbage, are an example of a land sharing approach which can be more productive than pasture alone and which confers high levels of welfare to farmed and wild animals whilst at the same time improving human and environmental welfare (152). At the other end of the spectrum the “high tech” Kipster farm produces One Welfare friendly (carbon neutral) eggs in a system that employs “low-opportunity-cost feedstuffs” (156). Clearly, there is not a “one size fits all” solution (153, 157). We must create context specific solutions, which are best developed by rigorous collaboration between disciplines (158), which consider individuals, communities, populations, and ecosystems (159) and which are supported by connected research, incentives, and political will (135).

COVID-19 raised a myriad of One Welfare concerns associated with livestock production. In so doing it has highlighted the fractures in our current food system like no other challenge before. Our fragile food system requires urgent and radical change to build resilience and ensure food security in the face of future challenges, including climate change. Fortunately, COVID-19 also presents us with a unique opportunity for a One Welfare driven transformation of the food production system. This will ensure a resilient, safer, fairer, and potentially healthier environment for both humans and animals in the future.

## Author Contributions

JM-F and LB participated equally in the conception, writing, and refining of the article.

## Conflict of Interest

The authors declare that the research was conducted in the absence of any commercial or financial relationships that could be construed as a potential conflict of interest.
