# Corrigendum: COVID-19 Effects on Livestock Production: A One Welfare Issue

**DOI:** 10.3389/fvets.2020.625372

**Published:** 2020-11-19

**Authors:** Jeremy N. Marchant-Forde, Laura A. Boyle

**Affiliations:** ^1^United States Department of Agriculture - Agricultural Research Service, Livestock Behavior Research Unit, West Lafayette, IN, United States; ^2^Pig Development Department, Teagasc Animal and Grassland Research and Innovation Centre, Fermoy, Ireland

**Keywords:** poultry, pigs, livestock production chain, one welfare, COVID-19

In the original article, there was an error. **An incomplete disclaimer was used**.

A correction has been made to ***Disclaimer***:

**Disclaimer:** The findings and conclusions in this publication are those of the author(s) and should not be construed to represent any official USDA or U.S. Government determination or policy. Mention of any trade name, proprietary product or specific equipment does not constitute a guarantee or warranty by USDA-ARS and does not imply its approval to the exclusion of other products that may also be suitable. The USDA-ARS is an equal opportunity and affirmative action employer and all agency services are available without discrimination.

The authors apologize for this error and state that this does not change the scientific conclusions of the article in any way. The original article has been updated.

